# The HIV-1 Reverse Transcriptase A62V Mutation Influences Replication Fidelity and Viral Fitness in the Context of Multi-Drug-Resistant Mutations

**DOI:** 10.3390/v10070376

**Published:** 2018-07-19

**Authors:** José O. Maldonado, Louis M. Mansky

**Affiliations:** 1 Institute for Molecular Virology & DDS-PhD Dual Degree Program, University of Minnesota-Twin Cities, 18-242 Moos Tower, 515 Delaware Street SE, Minneapolis, MN 55455, USA; jmaldo@umn.edu; 2 Institute for Molecular Virology, University of Minnesota-Twin Cities, 18-242 Moos Tower, 515 Delaware Street SE, Minneapolis, MN 55455, USA

**Keywords:** lentivirus, immunodeficiency virus, viral fitness, viral mutagenesis, compensatory mutation

## Abstract

Emergence of human immunodeficiency virus type 1 (HIV-1) drug resistance arises from mutation fixation in the viral genome during antiretroviral therapy. Primary mutations directly confer antiviral drug resistance, while secondary mutations arise that do not confer drug resistance. The A62V amino acid substitution in HIV-1 reverse transcriptase (RT) was observed to be associated with multi-drug resistance, but is not known to be a resistance-conferring mutation. In particular, A62V was observed in various multi-dideoxynucleoside resistant (MDR) mutation complexes, including the Q151M complex (i.e., A62V, V75I, F77L, F116Y, and Q151M), and the T69SSS insertion complex, which has a serine–serine insertion between amino acid positions 69 and 70 (i.e., M41L, A62V, T69SSS, K70R, and T215Y). However, what selective advantage is conferred to the virus remains unresolved. In this study, we hypothesized that A62V could influence replication fidelity and viral fitness with viruses harboring the Q151M and T69SSS MDR mutation complexes. A single-cycle replication assay and a dual-competition fitness assay were used to assess viral mutant frequency and viral fitness, respectively. A62V was found to increase the observed lower mutant frequency identified with each of the viruses harboring the MDR mutation complexes in the single-cycle assay. Furthermore, A62V was observed to improve viral fitness of replication-competent MDR viruses. Taken together, these observations indicate an adaptive role of A62V in virus replication fidelity and viral fitness, which would likely enhance virus persistence during drug-selective pressure.

## 1. Introduction

Since the discovery of the human immunodeficiency virus type 1 (HIV-1), the etiological agent of acquired immunodeficiency syndrome (AIDS), in the early 1980s, approximately 30 million individuals have died of AIDS, and approximately 36 million are currently infected worldwide. Current treatment for HIV-1 infection consists of a combination drug therapy (highly-active antiretroviral therapy, HAART) [1]. HAART typically consists of nucleoside reverse transcriptase inhibitors (NRTIs) [2], non-nucleoside reverse transcriptase inhibitors (NNRTIs) [3], and protease inhibitors (PIs) or integrase inhibitors which target key steps of the viral life cycle. HAART dramatically reduced the rate of HIV-1 and AIDS-related morbidity and mortality [4,5]. A key drawback of drug therapy is antiretroviral drug resistance, which is associated with the acquisition of drug-resistant mutations [6]. The high mutation rate of HIV-1 (i.e., 3.4 × 10^−5^ mutations per target bp per replication cycle) [7] arguably contributes to the evolution of drug resistance. This high mutation rate can, in principle, lead to the generation of viral genomes, each day, which possess every possible mutation within an infected individual, thus allowing for the selection of drug-resistant mutations from this population [8]. Selection of virus variants with drug-resistant mutations that confer greater fitness for replication emerge during selective drug pressure. Fitness is defined as a parameter describing the capacity of an organism to adapt and replicate in a given environment (reviewed in Reference [9]). Two general types of mutations associated with drug-resistant phenotypes are (1) primary mutations that confer direct drug resistance, and (2) secondary mutations that emerge during continued drug-selective pressure [10]. The former types of mutations usually correlate with a decrease in viral fitness, while the latter may have no discernable phenotype, but could include adaptive mutations that improve fitness. Latently infected cells can harbor drug-resistant viruses [11,12], which are an obstacle to antiretroviral therapy. Understanding HIV-1 population dynamics in the context of drug-resistant mutations is essential to better predict viral disease progression to AIDS, durable antiretroviral drug regimens, and vaccine development.

HIV-1 reverse transcriptase (RT), which is the enzyme responsible for converting the single-stranded viral RNA genome into double-stranded DNA, was previously observed to acquire the A62V amino acid substitution, which is known to be associated with multi-drug resistance but is not a resistance-conferring mutation [13,14]. In particular, A62V is normally seen in different mutational arrangements, located mostly on the flexible β3–β4 loop region of the fingers sub-domain of HIV-1 RT, including the multi-dideoxynucleoside resistant (MDR) Q151M complex (i.e., A62V, V75I, F77L, F116Y, and Q151M) [13,14] and the T69SSS insertion complex, which has a serine–serine insertion between the amino acid positions 69 and 70 (i.e., M41L, A62V, T69SSS, K70R, and T215Y) [15,16,17]. Mutational insertions or deletions in the β3–β4 loop region may confer multi-drug resistance [18]. The Q151M complex and the T69SSS insertion complex confer resistance to most of the NRTIs currently used for treatment including didanosine, zalcitabine, stavudine, and zidovudine (AZT), while the Q151M complex additionally confers resistance to lamivudine and abacavir [17,18,19]. These MDR complexes may lead to higher mortality rates, and can be transmitted from mother to child [20,21]. An initial study reported that the A62V mutation alone increases HIV-1 mutant frequencies, and causes a minor decrease in virus fitness [22].

In this study, we sought to determine the role of A62V in HIV-1 viral mutagenesis and fitness in the context of clinically relevant drug-resistant mutations. To do this, we first used a single-cycle assay to assess the mutant frequency of the HIV-1 MDR complex mutants (i.e., the Q151M complex and the T69SSS insertion complex) in the presence or absence of the A62V substitution. The inclusion of the A62V substitution increased the observed lower mutant frequencies with each of the HIV-1 MDR complex mutants. We also introduced these mutations into an HIV-1 NL4-3 infectious molecular clone, and assessed its impact on viral fitness. Notably, the inclusion of A62V in the context of various MDR mutation complexes improved viral fitness in the presence of AZT. These observations together implicate an adaptive role for A62V in enhancing virus persistence during drug-selective pressure.

## 2. Materials and Methods

### 2.1. HIV-1 Vectors and Cell Lines

**Figure 1:**
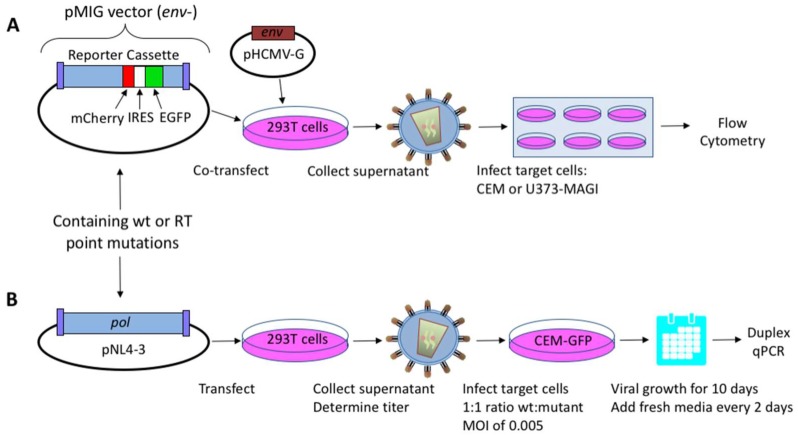
Assays for measuring human immunodeficiency virus type 1 (HIV-1) mutant frequencies and replication capacity. (**A**) Mutant frequency assay. 293T cells were co-transfected with an HIV-1 vector containing the dual-reporter mCherry and enhanced GFP (EGFP) cassette (pNL4-3 MIG) and a vesicular stomatitis virus G (VSV-G) envelope expression plasmid (pHCMV-G) in order to produce pseudotyped vector virus. Cell culture supernatants were collected and used to infect permissive CEM or U373-MAGI cells. Determination of virus mutant frequencies and replication capacities under zidovudine (AZT)-selective pressure were done using U373-MAGI cells. Relative infectivity and mutant frequencies were determined by flow cytometry. (**B**) Dual competition assay. Mutations in HIV-1 reverse transcriptase (RT) were introduced in an infectious molecular clone (pNL4-3), and were used to produce infectious virus following transfection of proviral DNA into 293T cells. Mutant and wild-type (wt) viruses were used to co-infect 5 × 10^5^ permissive CEM-EGFP target cells at a 1:1 ratio, using a multiplicity of infection (MOI) of 0.005. Infected target cells were maintained for 10 days by replenishing with fresh media every other day. Cells were then collected, genomic DNA extracted, and relative amounts of viral nucleic acid quantified by duplex qPCR. Abbreviations: IRES, internal ribosome entry site; *pol*, HIV-1 gene consisting of protease, RT, and integrase.

The HIV-1 dual-reporter vector, pNL4-3 MIG, which expresses mCherry and the enhanced green fluorescent protein (EGFP), was previously described [23,24]. This HIV-1 vector was co-transfected with a vesicular stomatitis virus G protein (VSV-G) envelope expression plasmid (HCMV-G; kindly provided by J. Burns, University of California, San Diego, CA, USA), into 293T cells to produce an infectious vector virus for use in a single-cycle replication assay (Figure 1A). HIV-1 NL4-3 molecular clones with polymorphisms in the *vif* gene (a kind gift from E. Arts, Case Western Reserve University, Cleveland, OH, USA) were used in virus fitness assays as previously described [22,25]. Briefly, the HIV-1 RT mutants analyzed in this study were inserted into HIV-1 *pol*, with the 2100–5983 base region from HIV-1 NL4-3 sub-cloned into pCR2.1-TOPO^®^ (Invitrogen, Carlsbad, CA, USA). Site-directed mutagenesis (QuikChange II Site-Directed Mutagenesis; Stratagene, Santa Clara, CA, USA) was performed to introduce point mutations; the region was sequenced to confirm proper introduction of mutations, and was then cloned back into the pNL4-3 MIG vector, using SbfI (2844) and AgeI (3486) restriction enzyme sites (New England Biolabs, Ipswich, MA, USA), or into the NL4-3 molecular clone using MscI restriction enzyme sites (2683 and 4545). All clones were sequence-confirmed for orientation and presence of desired mutations.

Human embryonic kidney (HEK 293T) cells (American Type Culture Collection, Manassas, VA, USA) were maintained in Dulbecco’s modified Eagle’s medium (DMEM; Cellgro, Manassas, VA, USA) plus 10% FetalClone III (FC3; Hyclone, Thermo Scientific, Waltham, MA, USA) and 1% penicillin/streptomycin (Invitrogen, Carlsbad, CA, USA). U373-MAGI-CXCR4 cells were obtained from Michael Emerman through the National Institutes of Health (NIH) AIDS Reagent Program, Division of AIDS, NIAID, NIH [26]. U373-MAGI cells were maintained as were the HEK 293T cells, but also included the addition of 1.0 µg/mL puromycin, 0.1 mg/mL hygromycin B, and 0.2 mg/mL neomycin. The CEM-EGFP cell line was obtained from the AIDS Research and Reference Reagent Program, contributed by J. Corbeil [27]. The CEM cell line was a kind gift from Michael Malim. The CEM and CEM-EGFP cell lines were maintained in Roswell Park Memorial Institute (RPMI) medium (Gibco, Life Technologies Invitrogen, Grand Island, NY, USA) plus 10% FC3.

### 2.2. Virus Production and Titer Assay

Vector viruses from the NL4-3 MIG vector and infectious viruses from the NL4-3 molecular clone were produced via transient transfection of HEK 293T cells as previously described [22] (Figure 1B). Briefly, the polyethylenimine (PEI) method [28] was used to transfect DNA into 2 × 10^6^ 293T cells with 10 μg of vector virus/proviral plasmid DNA, 1 μg of HCMV-G envelope expression plasmid DNA, and 33 µL of 1 mg/mL PEI. The medium was replaced 18 h post-transfection, and the cell culture supernatants were collected 48 h post-transfection, before being filtered through a 0.2-μm filter.

A tissue culture infectious dose (50%; TCID_50_) end-point dilution assay was used to determine infectious units (IU) of virus per milliliter of cell culture supernatant. Each cell culture supernatant (for wild-type (wt) and mutant viruses) was serially diluted 10-fold; 100 μL of diluted supernatant was added to 5 × 10^4^ CEM-EGFP indicator cells in 250 μL of total volume, before being plated in a 96-well plate, *n* = 6. The media was replaced every 48 h, and, at day 10, the number of EGFP-positive wells was determined using fluorescence microscopy. The number of EGFP-positive wells was multiplied by 1/6 and summed with 0.5 to determine the TCID_50_. The TCID_50_ divided by 100 μL was defined as the equivalent of the IU in each milliliter of supernatant. For example, an MOI of 0.005 could then be computed by calculating the amount of supernatant with 50 IU (MOI = IU/number of cells; 0.005 × 10,000 cells = 50 IU).

The titer of virus stocks was determined using U373-MAGI cells prior to drug treatment experiments as previously described [23]. Briefly, a 12-well plate was used to plate 62,500 cells/well the day before infection. The media was replaced 18 h after plating the cells, and varying amounts of virus ranging from 1 to 50 µL were added. The media was replaced 24 h post-infection, and cells were collected 72 h post-infection. The cells were then analyzed by flow cytometry as previously described [29]. Briefly, the infected target cells were washed in phosphate-buffered saline (PBS) and resuspended in 200 µL of 2% FC3-PBS. Expression of mCherry and EGFP was analyzed using a BD LSR II flow cytometer (BD Biosciences, San Jose, CA, USA). Gates were selected based on a forward scatter channel and a side scatter channel with a minimum of 10,000 gated cells per sample. The fluorescent reporter proteins were excited with a blue 488-nm laser and a 561-nm laser, respectively. Flow cytometry data were analyzed using the FlowJo (v.9.2) software (Ashland, OR, USA). Virus infectivity was determined by adding all positive quadrants of mCherry-positive (mCherry^+^) and EGFP-positive (EGFP^+^) cells, and was set relative to wt for each experimental replicate.

### 2.3. Mutant Frequency Analysis by Flow Cytometry

Vector viruses were used to infect 5 × 10^4^ CEM cells via spinoculation for 2 h at 1200× *g*. Viral stocks were then titered using CEM cells to maintain a transduction efficiency below 20–30% to limit the likelihood of co-infection. Experiments were conducted independently three to five times with six biological replicates. The cells were prepared and analyzed by flow cytometry as described above. Mutant frequencies were calculated by dividing the sum of the number of cells in the single-positive populations (i.e., mCherry^+^, EGFP^−^ and mCherry^−^, EGFP^+^) by the total number of infected cells. The mutant frequencies were then set relative to wt for each experimental replicate.

### 2.4. Dual-Competition Assay

Infections using RT variants were done in the presence of the isogenic wt NL4-3 clone. In each head-to-head competition assay, the wt and mutant viruses could be independently quantified using a qPCR assay based on specific polymorphisms (i.e., 11 synonymous mutations) in the *vif* gene. Dual infections of 5 × 10^5^ CEM-EGFP cells were done with a 1:1 ratio of wt:mutant virus at an MOI of 0.005. The cultures were maintained for 10 days, adding fresh culture media every two days. Each pairwise competition was done independently three times with four biological replicates per competition.

### 2.5. TaqMan Duplex qPCR Assay

Analysis of dual-competition experiments was done using a modified duplex qPCR assay as previously described [25]. Briefly, infected cells were collected on day 10 of the competition assay, and then resuspended in 200 μL of PBS, before the total genomic DNA was extracted using the ZymoBead™ Genomic DNA Kit (Zymo Research, Irvine, CA, USA), and eluted into a total volume of 35 μL. Next, 5 μL of extracted DNA was subjected to a brief PCR amplification reaction in a 50-μL total-volume reaction with an outer primer pair—i.e., Vif Out^+^ (5′–GCA AAG CTC CTC TGG AAA GGT GAA GGG–3′) and Vif Out^−^ (5′–CTT CCA CTC CTG CCC AAG TAT CCC–3′) primers to amplify the HIV-1 *vif* gene. Reactions were performed with Platinum PCR Supermix (Invitrogen) under the following conditions: one cycle at 94 °C for 2 min; 10 cycles at 94 °C for 30 s, 55 °C for 30 s, and 68 °C for 45 s; and one cycle at 68 °C for 5 min. The reaction was then purified with a GenElute PCR Clean-Up Kit (Sigma), and was eluted to 35 μL.

A single probe was used in the TaqMan qPCR assay to differentiate between the two *vif* polymorphisms (i.e., vifA and vifB) as previously described [30]. One common-sense primer sequence was used for both vifA and vifB variants (vifAB; 5′–GGT CTG CAT ACA GGA GAA AGA GAC T–3′), and vifA- and vifB-specific antisense primers were used to differentially quantify the presence of each sequence. The vifA antisense primer used was 5′–AGG GTC TAC TTG TGT GCT ATA TCT CTT TT–3′, and the vifB antisense primer was 5′–AGG AAG CTT GCA ATA TCT AGC GTT AGC A–3′. The single probe (5′–6-FAM–CCT CCA TTC TAT GGA GAC TCC CTG ACC–BHQ1–3′), which was used for both vifA and vifB, was labeled with fluorescein (FAM) and Black Hole Quencher. All amplifications were done using a BioRad CFX96 Touch Real-Time PCR Detection System (BioRad, Hercules, CA, USA). Primers were optimized, and were added at a final concentration of 375 nM each, while the probe was added at a concentration of 250 nM. Next, 1 μL of PCR product was added to 10 µL of 2× iTaq™ Universal Probes Supermix (BioRad), and was adjusted to a 20-μL total reaction volume. Reaction conditions were as follows: 95 °C for 10 min, 95 °C for 15 s, and 53 °C for 1 min for 40 cycles. All reactions were performed in triplicate, including a 9-log range in the plasmid DNA template (i.e., 5 × 10^9^ to 5 × 10^1^ copies), as well as a no-template negative control. The PCR efficiency (E) of each standard curve (vifA or vifB) was calculated based on the curve slope, E = (10 − 1/s − 1) × 100%, and the standard curves were confirmed to be within 5%, while the R^2^ of each standard curve was ≥99% to maximize sample data confidence. Differences in relative amounts of each clone after appropriate viral growth were used to identify differences in virus fitness.

### 2.6. Calculation of Viral Fitness

Fitness differences (WD) were calculated for each wt versus mutant competition assay. The complementary DNA (cDNA) copy numbers of each of the four biological replicates used in the experiment were used to calculate the relative viral fitness (*d*) using the Viral Growth Rate Calculator web tool (https://indra.mullins.microbiol.washington.edu/vgrc/) [30]. Data from the three experimental replicates were compiled to determine fitness differences.

### 2.7. Statistical Analyses

All statistical analyses were done using the GraphPad Prism version 6.0 software (La Jolla, CA, USA). A one-way ANOVA statistical analysis was performed on the raw data prior to normalization, in order to determine differences between the wt and mutants for both mutant frequency and viral fitness. A Tukey’s multiple-comparison post-test was also performed, in order to compare differences between the wt and each mutant. Linear regression was used to identify R^2^ values.

## 3. Results

### 3.1. Mutant Frequency Analysis of HIV-1 RT Variants

**Figure 2:**
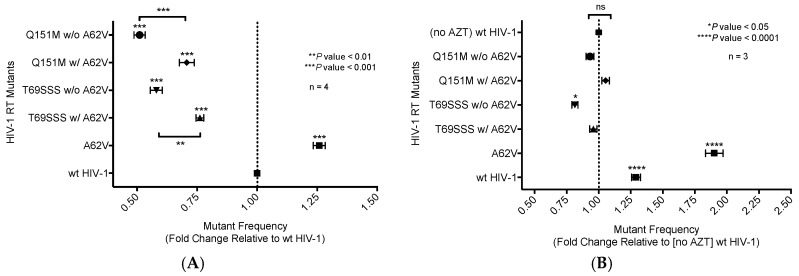
Mutant frequency analysis of multi-drug-resistant HIV-1 mutants in the presence or absence of A62V. Mutant frequency analysis was performed using the pNL4-3 MIG vector by infecting permissive target cells prior to analyzing for expression of a pair of marker genes (mCherry and EGFP) by flow cytometry. Mutant frequencies were calculated by dividing the sum of the number of cells in the single-positive populations (i.e., mCherry^+^, EGFP^−^ and mCherry^−^, EGFP^+^) by the total number of infected cells. The mutant frequencies were then set relative to the no-AZT wt virus for each experimental replicate. The A62V mutation in HIV-1 RT was analyzed alone or in the presence or absence of the multi-dideoxynucleoside resistant (MDR) Q151M complex (i.e., A62V, V75I, F77L, F116Y, and Q151M) and the T69SSS insertion complex (i.e., M41L, A62V, T69SSS, K70R, and T215Y). The mutant frequency of the MDR mutants, in the absence of AZT (**A**) or in the presence of AZT (**B**). The standard error of the mean (SEM) is represented by error bars. * *p*-value < 0.05; ** *p*-value < 0.01; *** *p*-value < 0.001; **** *p*-value < 0.0001. Lack of statistical significance is denoted by ‘ns’.

A single-cycle vector assay (Figure 1A) was used to analyze differences in mutant frequency (Figure 2). In this assay, all four MDR mutants (i.e., Q151M complex with or without A62V, and the T69SSS insertion complex with or without A62V) were found to have a lower mutant frequency relative to that of the wt virus in the absence of AZT (Figure 2A). Interestingly, the MDR mutants without the A62V mutation had the lowest observed mutant frequencies (i.e., roughly half that of HIV-1 wt), which was significantly different from those of MDR complex mutants, including the A62V mutation. The observed virus mutant frequency of the A62V mutant alone was the highest (1.25-fold) of all mutants tested relative to the wt virus, as anticipated based upon previous observations [22]. The changes in mutant frequency of the MDR mutants were then tested in the presence of AZT (Figure 2B). The mutant frequency of the MDR mutants (except for the T69SSS complex without A62V, which was significantly lower) was restored to wt levels under the pressure of AZT. The mutant frequency of virus variants harboring the A62V alone was further increased (i.e., 1.9-fold) in the presence of AZT relative to the wt virus in the absence of AZT. Of note, wt HIV-1 replication in the presence of AZT resulted in a 1.26-fold increase in mutant frequency.

### 3.2. Replication Capacity Analysis of HIV-1 RT Variants

**Figure 3:**
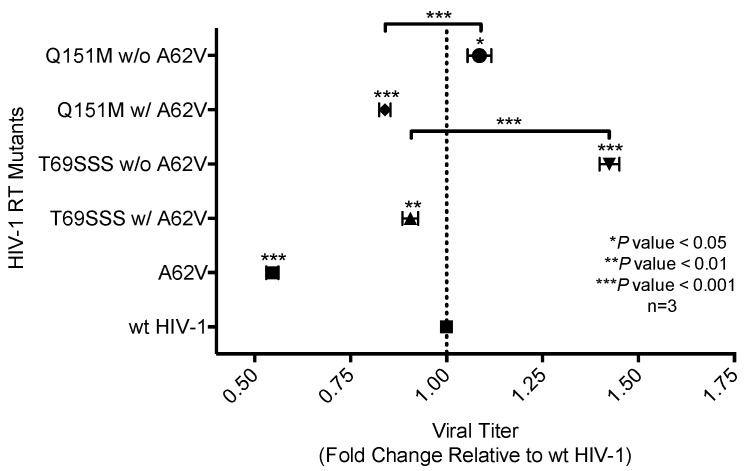
Analysis of replication capacity of multi-drug-resistant HIV-1 mutants in the presence or absence of A62V. Normalized virus stocks were used in the single-cycle assay to determine the replication capacity of multi-dideoxynucleoside resistant (MDR) HIV-1 vector viruses, the Q151M complex (i.e., A62V, V75I, F77L, F116Y, and Q151M) and the T69SSS insertion complex (i.e., M41L, A62V, T69SSS, K70R, and T215Y), in the presence or absence of A62V. Replication capacity was determined via the detection of GFP expression by flow cytometry. All replication capacity values were set relative to wt HIV-1 for each experimental replicate. The standard error of the mean (SEM) is indicated by the error bars. * *p*-value < 0.05; ** *p*-value < 0.01; *** *p*-value < 0.001.

**Figure 4:**
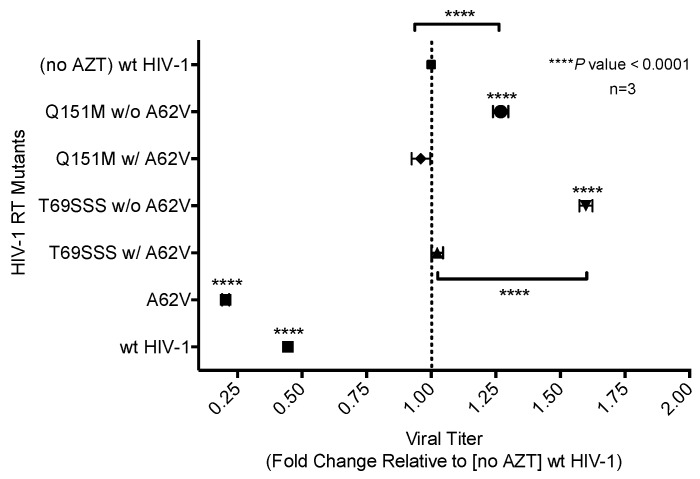
Analysis of relative drug susceptibility of multi-drug-resistant HIV-1 mutants in the presence or absence of A62V. Normalized virus stocks were used in the single-cycle assay to determine the relative drug susceptibility of the multi-dideoxynucleoside resistant (MDR) HIV-1 Q151M mutant complex (i.e., A62V, V75I, F77L, F116Y, and Q151M), and the T69SSS insertion complex (i.e., M41L, A62V, T69SSS, K70R, and T215Y) in the presence or absence of A62V under AZT-selective pressure. Virus titers were determined by flow cytometry. All drug susceptibility values were set relative to no-AZT wt HIV-1 for each experimental replicate. The standard error of the mean (SEM) is indicated by the error bars. **** *p*-value < 0.0001.

The single-cycle assay was used with normalized virus stocks to measure the expression of EGFP in infected cells to determine wt or mutant HIV-1 replication capacity (Figure 3), or drug susceptibility (Figure 4) in the absence or presence of AZT. The number of infected cells, as determined by flow cytometry, directly represented virus replication [31]. Two of the MDR complex mutants—the Q151M complex without A62V, and the T69SSS complex without A62V—each had an improved replication capacity compared to that of the wt virus, with the T69SSS complex without A62V mutant having the highest increase in replication capacity (Figure 3). In contrast, the two MDR complexes with A62V, as well as the A62V mutant, had a reduced replication capacity compared to that of wt virus (Figure 3). The A62V mutant was observed to have the lowest replication capacity of the viruses analyzed.

### 3.3. Drug Susceptibility of HIV-1 RT Variants to AZT

AZT drug susceptibility among the HIV-1 RT variants compared to the wt was analyzed by determining the IC_50_ values. The A62V mutant was observed to have the highest susceptibility to AZT with a 1.76-fold decrease in virus replication relative to that of the wt virus in the absence of AZT. The two MDR complex mutants without A62V were observed to have the highest level of drug susceptibility, with the T69SSS insertion complex having the highest fold increase (Figure 4). The Q151M complex and T69SSS insertion complex mutants with A62V had an AZT drug-susceptibility phenotype comparable to that of the wt virus.

### 3.4. Fitness Impact of HIV-1 RT Mutations

**Figure 5:**
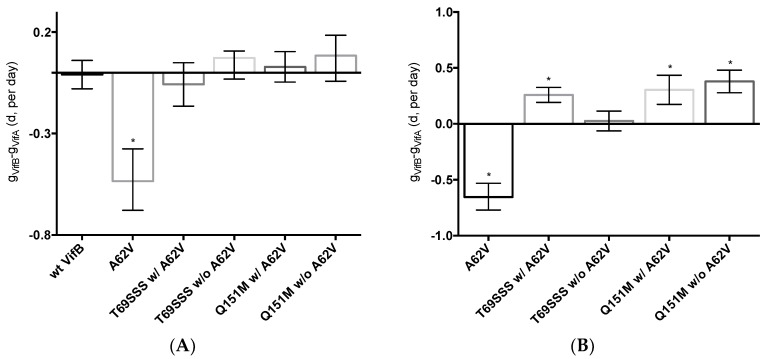
Dual-competition virus fitness assay of multi-drug-resistant HIV-1 mutants in the presence or absence of A62V. The net growth rate difference (*d*) of multi-dideoxynucleoside resistant (MDR) HIV-1 mutants (with or without A62V), in the absence (**A**) or presence (**B**) of AZT, was determined using the difference between the growth rate of wt HIV-1 (vifB) and mutant (vifA), that is, g_VifB_ − g_VifA_. To assess the replication fitness, a real-time TaqMan PCR assay was used to monitor and differentiate between the mutant and parental virus using polymorphisms in the *vif* gene. All growth difference values (i.e., panels **A**,**B**) were set relative to no-AZT wt HIV-1 (*vifB*), shown in panel (**A**)*.* The polymorphisms in the *vif* gene had a small fitness impact, as shown in panel (**A**); this effect was taken into consideration for all calculations. The wt HIV-1 (*vifB*) was not directly included in panel (**B**) due to inability to replicate it in the presence of AZT. Reported are the means and 95% confidence intervals (CIs) from triplicate competition experiments. The asterisk (*) indicates a significant difference (*p*-value < 0.05, calculated using methods explained elsewhere [30]) relative to the corresponding *vif* gene polymorphisms (*vifA*, mutant virus; *vifB*, parental virus).

To assess the replication fitness of HIV-1 harboring the MDR RT mutants, a real-time TaqMan PCR assay was used to monitor the mutant and parental viruses as previously described [30]. This method allowed for the net growth rate of each virus in competition assays to be determined. The net growth rate difference (*d*) per day (which was used to determine the fitness cost between the mutant and its parental virus) was calculated in the absence (Figure 5A) or presence (Figure 5B) of AZT. In the absence of AZT, the A62V mutant alone had significantly reduced viral replication fitness, while the T69SSS insertion complex with A62V had a non-statistically significant decrease in fitness (Figure 5A). The fitness differences of the Q151M complex and T69SSS insertion complex mutants without A62V were not significant. In the presence of AZT, all MDR mutant complexes, except for the T69SSS insertion complex without A62V, had a statistically significant increase in virus fitness (Figure 5B). Predictably, the A62V mutant alone had reduced fitness.

## 4. Discussion

**Figure 6:**
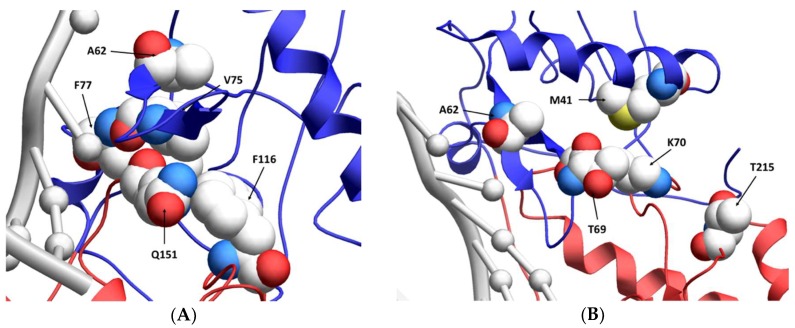
Location of resistance-conferring amino acid residues in multi-drug-resistant HIV-1 reverse transcriptase. A ribbon structure of the covalently trapped catalytic complex of HIV-1 RT [34] with the multi-dideoxynucleoside resistant (MDR) Q151M complex amino acid residues shown in a close-up view (**A**). The MDR T69SSS insertion complex amino acid residues are shown in a close-up view (**B**). A portion of the HIV-1 RT p66 subunit is shown with color-coding of subdomains: fingers (blue) and palm (red). The template and primer DNA strands are shown in light gray. While the p66 subunit contains the DNA-binding groove and the polymerization active site, the non-catalytic p51 subunit is not shown [35]. Image obtained from the “Research Collaboratory for Structural Bioinformatics Protein Data Bank” [36] (Protein Data Bank identifier: 1RTD).

While the A62V amino acid substitution in HIV-1 RT is known to be associated with multi-drug resistance, it is not a resistance-conferring mutation, and its appearance remains an open question in the field. To investigate this, we tested the hypothesis that A62V provides a selective advantage to the virus in the context of multi-drug resistance by influencing replication fidelity and fitness. In particular, we used parallel analyses to look at the relationship between HIV-1 fitness and mutagenesis in the presence or absence of the RT A62V amino acid substitution. We first found that the A62V mutation alone could significantly increase viral mutant frequencies (Figure 2) [22], while negatively impacting replication capacity (Figure 3 and Figure 4) and viral fitness (Figure 5) in the absence or presence of AZT. Both the Q151M complex and the T69SSS insertion complex share the A62V secondary drug-resistance-associated point mutation, which is located close to the active polymerization site of HIV-1 RT (Figure 6A,B, respectively). We observed that MDR mutants without the A62V mutation had the lowest mutant frequencies, suggesting that these RT variants have higher fidelity (Figure 2A). In the presence of AZT (Figure 2B), the mutant frequency of all the MDR viruses, except that of the T69SSS insertion complex without A62V, were restored to that of wt HIV-1 in the absence of AZT. These observations support the conclusion that the A62V mutation plays an important role in RT fidelity by increasing mutant frequency (Figure 2), where mutant frequency is higher in the context of MDR complexes in the absence of AZT (Figure 2A), but highest in virus with the A62V mutation alone in the presence of AZT (Figure 2B). How A62V influences replication is currently unclear, and will be a topic of future analyses.

The T69SSS insertion complex without A62V was observed to possess the highest replication capacity. In contrast, the two MDR variant viruses with A62V, along with the virus harboring the A62V mutant alone, had a lower replication capacity relative to wt HIV-1 (Figure 3). Under AZT-selective pressure, viruses harboring either the Q151M complex or the T69SSS insertion complex without A62V had the lowest level of drug susceptibility. In contrast, viruses harboring the Q151M complex or the T69SSS insertion complex in the context of A62V had replication efficiencies comparable to that of wt HIV-1 in the absence of a drug when under AZT drug-selective pressure (Figure 4). Taken together, these data support the conclusion that both MDR variant viruses (i.e., harboring the Q151M complex and the T69SSS insertion complex mutations), in the context of the A62V amino acid substitution, had mutant frequencies and replication capacities, when under AZT selective pressure, comparable to that of the wt virus in the absence of drug-selective pressure. These observations implicate an interrelationship between HIV-1 fitness and mutation rate [22].

All multi-drug-resistant viruses were observed to have a statistically significant increase in viral fitness under AZT-selective pressure, except viruses harboring the T69SSS insertion complex without A62V (Figure 5B). As anticipated, viruses harboring the A62V mutation alone had the most significant reduction in replication capacity. These observations, together with the observations made of the relative susceptibility to AZT (Figure 4), indicate that the MDR viruses without A62V analyzed in our study have reduced AZT drug susceptibility at the expense of replication capacity. This was most notable with the viruses harboring the T69SSS insertion complex in the absence of the A62V mutation. As previously reported [32,33], we observed that the fitness of the Q151M complex, in the presence or absence of the A62V mutation, was not significantly different than that of the wt virus (Figure 5A). Together, these observations implicate A62V as an adaptive mutation arising via drug pressure.

The replicative capacity of viruses harboring the MDR Q151M complex, in the presence or absence of the A62V mutation, was observed to be comparable under AZT drug pressure (Figure 5B). However, viruses harboring the Q151M complex without A62V had a higher level of drug resistance than that of viruses possessing the Q151M complex with A62V (Figure 4), which agrees with previous reports [13,32,37]. Taken together, these observations support the conclusion that the T69SSS insertion complex does not confer a fitness advantage in the absence of AZT (Figure 5A), but does improve fitness in the presence of AZT (Figure 5B). This conclusion agrees with previous observations regarding the increased fitness associated with the T69SSS insertion complex during drug-selective pressure [38].

In summary, the observations made in this study provide the first demonstration that A62V is an important adaptive mutation in multi-drug-resistant viruses that impacts the interplay of replication fidelity, virus fitness and drug susceptibility. These data argue in support of the importance of adaptive mutations that can “piggyback” along with drug-resistant mutations to improve overall viral fitness during drug-selective pressure. The observations of this study complement previous observations where MDR viruses were observed to be more fit than the wt virus in the absence of a drug [33]. Differences observed between the findings presented in this study regarding particular phenotypes compared to those previously reported are likely due to biological differences that can exist among virus isolates and cell types used for the analyses of virus mutant frequency and viral fitness. These studies predict that, in general, viral mutation rate and fitness can be influenced by adaptive mutations that arise during drug-selective pressure.
